# Measuring and evolution analyzing of the reliability in urban public transport composite network

**DOI:** 10.1371/journal.pone.0340590

**Published:** 2026-01-08

**Authors:** Qingyu Luo, Haiyue Yu, Lili Yang, Tingting Gao

**Affiliations:** 1 Transportation College, Jilin University, Changchun, China; 2 FAW Group Co., Ltd, Changchun, China; Chang'an University, CHINA

## Abstract

With the expansion of urban public transport systems and the increasing integration of multiple modes, ensuring the reliability of composite public transport network has become a critical challenge. To overcome the limitations of traditional reliability research, which primarily relies on connec-tivity analysis, this paper proposes an innovative indicator system integrating connectivity reliability with a newly constructed transport capacity reliability dimension. The latter incorporates dynamic metrics such as passenger load and time delay to more comprehensively characterize actual operational conditions. By establishing a Coupled Map Lattice (CML) model to simulate the cascading propagation of node and line failures under multimodal coupling effects, this study reveals the failure mechanisms and recovery potential of complex networks under dynamic disturbances. Taking Changchun as a case study, the analysis reveals that both connectivity and transport capacity reliability experience minimal decline in the initial stages of disruption. When the scale of network damage exceeds 30%, transport capacity reliability shows a slight rebound. However, once the damage surpasses 50%, both indicators approach zero. The findings suggest that isolating faulty nodes in the early stages and prioritizing the recovery of high-betweenness nodes in the mid-stage can help maintain network operational efficiency. These results provide important theoretical guidance for enhancing the operational reliability and service quality of public transport systems. By applying complex network theory and data-driven methodologies, this research contributes to improving the resilience and sustainability of urban transport systems.

## Introduction

Urban public transport systems are encountering a range of challenges: increasing congestion leads to reduced punctuality, unbalanced capacity allocation results in overcrowded lines, and the growing structural complexity of networks elevates the risk of failures. These issues severely compromise the reliability of urban public transport. As conventional bus routes and urban rail transit systems continue to expand into interconnected networks, they play crucial roles in handling large-scale commuting flows. Analyzing reliability from the perspective of network structure and transport efficiency enables a deeper understanding of the latent capacity of composite public transport networks, helps prevent failure risks, and assists transport authorities in enhancing network reliability.

Currently, the reliability analysis of public transport network still faces the following issues. Firstly, most reliability evaluations are limited to single subway networks or bus networks, without fully recognizing the interconnections within composite networks. These interconnections can sometimes manifest as complementary relationships, while at other times, they may lead to cascading failures. Secondly, existing reliability assessments are primarily based on physical topological structures and fail to account for dynamic reliability issues arising from varying passenger loads and fluctuations in vehicle operation times. This can result in a situation where, despite physical structural connections, the system becomes dynamically unsustainable. Moreover, reliability issues are not always influenced by a single factor. The coupled effects of multiple influencing factors also require in-depth analysis.

This study tries to solve the above problems by the following research.

1. To develops an integrated reliability evaluation framework for urban public transport composite networks accounting for the interactions between urban rail and bus systems.
2. To construct a dual-layer reliability system combining connectivity and transport capacity by introducing passenger flow and temporal indicators to capture the dynamic operational characteristics.
3. To model cascading failures by applying Coupled Map Lattice (CML) model to simulate the spatiotemporal evolution of disruptions with dynamic feedback.

The rest of the paper is organized as follows. Section 2 reviews the literature related to the study of transportation network reliability. Section 3 provides details of the measurement method and evolution analysis method for reliability of urban public transport composite network. Section 4 presents case analysis and simulation experiments. Finally, Section 5 summarizes the study and discusses future research directions.

## Literature review

Early research on transportation network reliability primarily focused on connectivity reliability. Numerous scholars identified key factors influencing reliability by analyzing the topological characteristics and statistical metrics of transportation networks. Connectivity reliability was initially proposed by Japanese scholars Kawai and Mine (1982) in the context of road networks. This concept solely examines network topology, neglecting critical factors such as route length, traffic volume, and throughput capacity. It employs a binary (0–1) metric to determine whether links or nodes are connected, assessing the probability of reliable connections between network nodes [1]. Regarding connectivity reliability assessment methods, researchers have endeavored to address challenges related to computational complexity and metric practicality. Fan et al. (2015) proposed the efficient Target Order algorithm for the NP-hard connectivity reliability problem, providing a feasible tool for evaluating large-scale real-world road networks [2]. Yang (2019) introduced a dynamic strongly connected reliability model based on Didi trajectory data, utilizing relative speed thresholds to screen connected segments and offering new insights for practical road network assessment [3]. Neila Bhouri et al. (2025) introduced four spatio-temporal reliability and accessibility metrics for public transit systems, providing quantitative foundations for optimized scheduling [4]. These methodological breakthroughs have also driven applications in complex scenarios involving multimodal transportation and multiple disasters. Leveraging efficient evaluation algorithms and metrics, Wang et al. (2023) constructed a global directed network for crude oil maritime transport, precisely identifying critical shipping routes and assessing their vulnerability [5]. In disaster response, Qin et al. (2022) and Chen et al. (2021) developed post-disaster recovery and pre-disaster protection optimization models that deeply integrate constraints such as connectivity and temporal reliability, significantly enhancing the resilience and response efficiency of emergency logistics networks [6,7]. Wu et al. (2018) established a provincial expressway connectivity evaluation system, providing support for regional transportation infrastructure planning and protection [8].

With the increasing diversity of urban transport resources, it has become evident that focusing solely on connectivity reliability is insufficient for improving overall network reliability. Therefore, Shariat et al. (2010) subdivided transportation network reliability into connectivity reliability, capacity reliability, and travel time reliability [9]; Chen et al. (2013) defined capacity reliability as the probability that a transportation network can accommodate a certain level of transport demand while maintaining an acceptable service level, highlighting its crucial role in reflecting the service quality and efficiency of freight transportation networks [10]. Regarding capacity reliability research methodologies, Fang et al. (2019) quantified the impact of service quality on network reserve capacity using a two-layer planning model based on segment service level constraints and random user equilibrium [11]. Yuan et al. (2023) improved directed weighted centrality metrics and proposed a cascading failure suppression mechanism prioritizing downstream node redundant capacity during load diversion [12]. These methodological innovations directly drive the deepening of multidimensional application scenarios, continuously unlocking new contexts from traditional road networks to multimodal transport and intelligent connected systems. Li (2024) employed a derivative cutset algorithm to compare road network structures, demonstrating that grid-like topologies exhibit lower capacity collapse under deliberate attacks than ring-radial networks due to their redundancy advantages [13]. Ma et al. (2018) combined network transformation methods with Monte Carlo experiments to achieve the first reliability assessment of integrated transport networks under random capacity fluctuations [14]. Hao et al. (2022) innovatively developed a CAV/HDV hybrid traffic flow allocation model for intelligent connected environments, revealing and confirming the significant enhancement of CAV technology on capacity stability [15].

Compared to extensive research on road network reliability, existing studies focusing on public transport network reliability account for only about 8% [16]. Liu et al. (2021) quantified urban rail transit network reliability as the proportion of successful trips and evaluated transport service quality by integrating average generalized trip cost with passenger completion rate [17]. In related work, Dong et al. (2023) introduced the concept of “link reliability” into road traffic performance analysis. By establishing link quality thresholds, they proposed a new perspective for assessing network resilience and provided theoretical support for analyzing the dynamic recovery capability of transportation networks under flood disruptions [18].

Regarding metrics for measuring transportation network reliability, most studies focus on capturing complexity indicators reflecting aggregate behavior and macro-topological features. For instance, Zhang et al. (2018) employed degree, degree distribution, clustering coefficient, and betweenness centrality as indicators for evaluating transfer reliability. They proposed an efficient method for calculating travel time between stations based on the accessibility matrix and assessed the transfer reliability of Jinan’s conventional bus network [19]. Zhou Jie et al. (2025) employed the spatial L-method to construct a multilayer transportation network. They established a comprehensive topological metric system encompassing node overlap rate, node activity level, average path length, network diameter, clustering coefficient, betweenness centrality, multilayer participation coefficient, edge overlap rate, and edge intersection coefficient to analyze network structure and reliability [20]. Chen Ming et al. (2022) proposed a novel metric—travel time and connectivity reliability—to evaluate bridge network reliability under seismic scenarios. This metric comprehensively considers post-earthquake changes in travel time and network connectivity, thereby enhancing the completeness and practicality of assessments [21].

To characterize the reliability evolution of urban transportation networks under various disturbance scenarios, recent studies increasingly adopt a cascading failure framework to reveal how localized disruptions escalate into large-scale systemic risks. Early studies primarily focused on attack strategies and failure propagation mechanisms, simulating disturbance conditions through random attacks or deliberate removal (e.g., targeting highly connected nodes or nodes with high betweenness centrality). These studies typically employed load-capacity models or seepage theory to simulate cascading processes. However, such models often rely on static network topologies and overly simplified disturbance assumptions, failing to capture the inherent dynamic feedback effects and coupled evolutionary behavior of multimodal transportation systems.

To overcome these limitations, the Coupled Meshwork (CML) model emerged as a nonlinear dynamical modeling framework capable of simulating time-varying states of nodes and edges under perturbations. The CML model is particularly well-suited for characterizing failure propagation and recovery pathways in multilayer transportation systems. For instance, Xu et al. (2025) investigated the reliability of multi-state supply chain networks (MSCNs) under capacity and emission constraints by modeling transport routes with multiple operational states to reflect real-world uncertainties [22]. Zhu et al. (2021) constructed a Shanghai metro system model based on CML, introducing flow-weighted reliability metrics to evaluate cascading failure processes under different scenarios [23]. Furthermore, Wang et al. (2025) proposed a weighted CML model integrating structural connectivity and transport capacity to simulate the progressive degradation and system response of urban rail transit under cascading disturbances [24].

More recently, the CML framework has been extended to incorporate multilayer network coupling, adaptive flow redistribution mechanisms, and dynamic recovery strategies, offering a more comprehensive and evolution-oriented modeling tool for simulating cascading failures in composite bus–rail transit systems.

In summary, the existing body of research presents the following key limitations.

1. Incomplete theoretical framework. Most studies lack a systematic reliability evaluation model and are confined to single-mode networks, failing to account for the synergistic effects between urban rail transit and conventional bus systems.
2. Limited evaluation indicators. Current metrics primarily focus on topological reliability while neglecting dynamic characteristics such as passenger flow distribution and travel delay, making it difficult to comprehensively reflect the actual operational state of the network.
3. Limited representation of cascading evolution mechanisms. Current approaches primarily initiate disruptions through static attack strategies without embedding the dynamic feedback and spatiotemporal propagation characteristics that define real-world cascading failures. This constrains the ability to model how localized failures escalate into large-scale systemic disruptions within composite transit networks.

## Methodology

### Measurement method for reliability of urban public transport composite network

#### Definition.

In this study, the reliability of urban public transport composite network is defined as the network’s ability to meet both connectivity and transport service requirements within a specified time and capacity constraint under various random or targeted attacks.

From the perspectives of complex network theory and data-driven modeling, the reliability of a composite public transport network can be decomposed into two components: connectivity reliability and transport capacity reliability. Connectivity reliability refers to the ability of the composite network to maintain inter-node connectivity under varying conditions, ensuring that alternative routes or transport modes are available to allow passengers to reach their destinations on time. Transport capacity reliability reflects the composite network’s ability to provide normal transit services within a required time frame, including the evacuation of passenger flows and the timely operation of bus and subway systems. The service quality and operational stability of the composite network are jointly characterized by indicators such as the spatiotemporal distribution of passenger flows and vehicle delay times, etc.

#### Metrics.

1. Network connectivity reliability

The network connectivity reliability of an urban public transport composite network is defined as the average ratio between the number of effective paths for each OD (Origin-Destination) pair before and after network disruption. The formula is as follow:


$$\rho_{ij}=\frac{{Q_{ij}}^{\prime }}{Q_{ij}}$$ (1)

$$\rho_{G}=\frac{\sum_{s=\text{1}}^{S}\rho_{ij}}{S}$$ (2)

Where $\rho_{ij}$ is the ratio of effective paths for the OD pair after and before attack, $Q_{ij}$ is the number of effective paths before attack, ${Q_{ij}}^{\prime }$ is the number after attack, *S* is the total number of OD pairs, $\rho_{G}$ is the overall connectivity reliability. The value of $\rho_{G}$ approaching 1 indicates high reliability. $\;S=\left\{(i,j)|Q_{ij}>\text{0}\right\}\;$ denotes the set of OD pairs that have effective paths in the initial network.

The number of effective paths for each OD pair can be obtained using a valid path search algorithm. By aggregating these values across all OD pairs, the total effective paths of the network can be determined.

2. Transport capacity reliability

Transport capacity reliability refers to the ability of the remaining network nodes to dissipate passenger flows and maintain punctual operation after partial network damage. It can be evaluated from two indicators: the weighted betweenness of network passenger flow and the standard deviation of network delay time [25].

- Passenger flow–weighted betweenness centrality

The passenger flow-weighted betweenness centrality reflects a node’s role in passenger flow dissipation. In an unweighted network, the standard betweenness centrality is defined as the number of shortest paths passing through a given node, which is often used to evaluate the centrality of node in the network. However, a node with high unweighted betweenness may not hold a central role in a weighted passenger network. Therefore, the passenger flow-weighted betweenness centrality [26] is defined as the product of normalized unweighted betweenness and actual daily passenger flow at that node.


$$C_{B}^{W}(V)=W_{V}C_{B}(v)=W_{V}\sum_{s,t\in V}\frac{\text{2}\theta \left(s,t|v\right)}{\left(n-\text{1})(n-\text{2})\theta (s,t\right)}$$ (3)

$$W_{V}=\frac{nQ_{v}}{\sum_{i=\text{1}}^{n}Q_{i}}$$ (4)

$$C(V)=\frac{\text{1}}{n}\sum_{v=\text{1}}^{n}\sum_{s,t\in V}W_{V}C_{B}(v)$$ (5)

The term $C_{B}^{W}(v)$ denotes the passenger flow weighted betweenness centrality of node v. Here, θ(s,t) represents the total number of shortest paths between nodes s and t, while θ(s,t|v) denotes the number of those shortest paths that pass through node v. When s=t, it is defined that θ(s,t)=1. The parameter n denotes the total number of nodes in the network, and V represents the set of all network stations. $Q_{v}$ indicates the actual number of passengers passing through node v in the daily passenger flow of the network. $W_{V}$ is defined as the ratio of $Q_{v}$ to the average passenger flow across all nodes in the network, which is used to measure the relative magnitude of passenger flow at a given node. C(V) denotes the overall passenger flow weighted betweenness centrality of the network. This metric reflects the capability of the network to redistribute passenger flows. A larger value of C(V) indicates that the composite network possesses higher reliability.

- In unweighted networks, nodes exhibiting high betweenness centrality do not necessarily maintain prominent hub status once actual passenger-flow weights are incorporated. Compared with simple indicators such as raw passenger volume or unweighted betweenness, passenger-flow-weighted betweenness provides a more discriminative measure by identifying nodes that are structurally critical yet carry relatively low passenger demand, thereby preventing the overestimation of the network’s effective transport capacity.Standard deviation of network delay time

Here, the Mahalanobis distance [26] is used to characterize temporal efficiency and reflect the reliability of transport service. It quantifies the deviation of observed delays from historical patterns, thus serving as an indicator of network performance.


$$\mu^{t}=\frac{\sum_{k}x^{k}}{\left|\varepsilon^{t}\right|}$$ (6)

$$Q^{t}=\frac{\left|\varepsilon^{t}\right|}{\left|\varepsilon^{t}\right|-\text{1}}\sum_{k}\left(\frac{x^{k}{\left(x^{k}\right)}^{T}}{\left|\varepsilon^{t}\right|}-\mu^{k}{\left(\mu^{k}\right)}^{T}\right)$$ (7)

$$M^{t}=\sqrt{{\left(x^{t}-\mu^{t}\right)}^{T}Q^{-\text{1}}\left(x^{t}-\mu^{t}\right)}$$ (8)

Assume that within a given time interval t, the network consists of *N* stations. The delay observation vector for this period is defined as $x^{t}={\left[{\overset{―}{D}}_{\text{1}}^{t},{\overset{―}{D}}_{\text{2}}^{t},\cdots ,{\overset{―}{D}}_{N}^{t}\right]}^{T}$, where $x^{k}$ denotes the *k*-th observation vector in the historical sample set. Let $\mu^{t}$ represent the mean vector, $\;\varepsilon^{t}$ denote the historical delay dataset of the network, and $Q^{t}$ be the corresponding covariance matrix. The Mahalanobis distance $M^{t}$ is then computed, where a smaller $M^{t}$ value indicates higher network reliability. The probabilistic nature of the Mahalanobis distance enables a more rigorous identification of anomalous observations in noisy multivariate data, compared to directly analyzing raw delay data and applying heuristic thresholds to detect abnormal delays.

In a connected network, a significant reduction in transport capacity at key nodes can severely impair service quality and operational efficiency, even though the topological connectivity is maintained. Therefore, integrating flow-weighted betweenness and delay-time standard deviation enables the identification of nodes that, while critical in terms of connectivity, exhibit relatively poor actual transportation capacity.

#### Measurement process.

**Fig 1:**
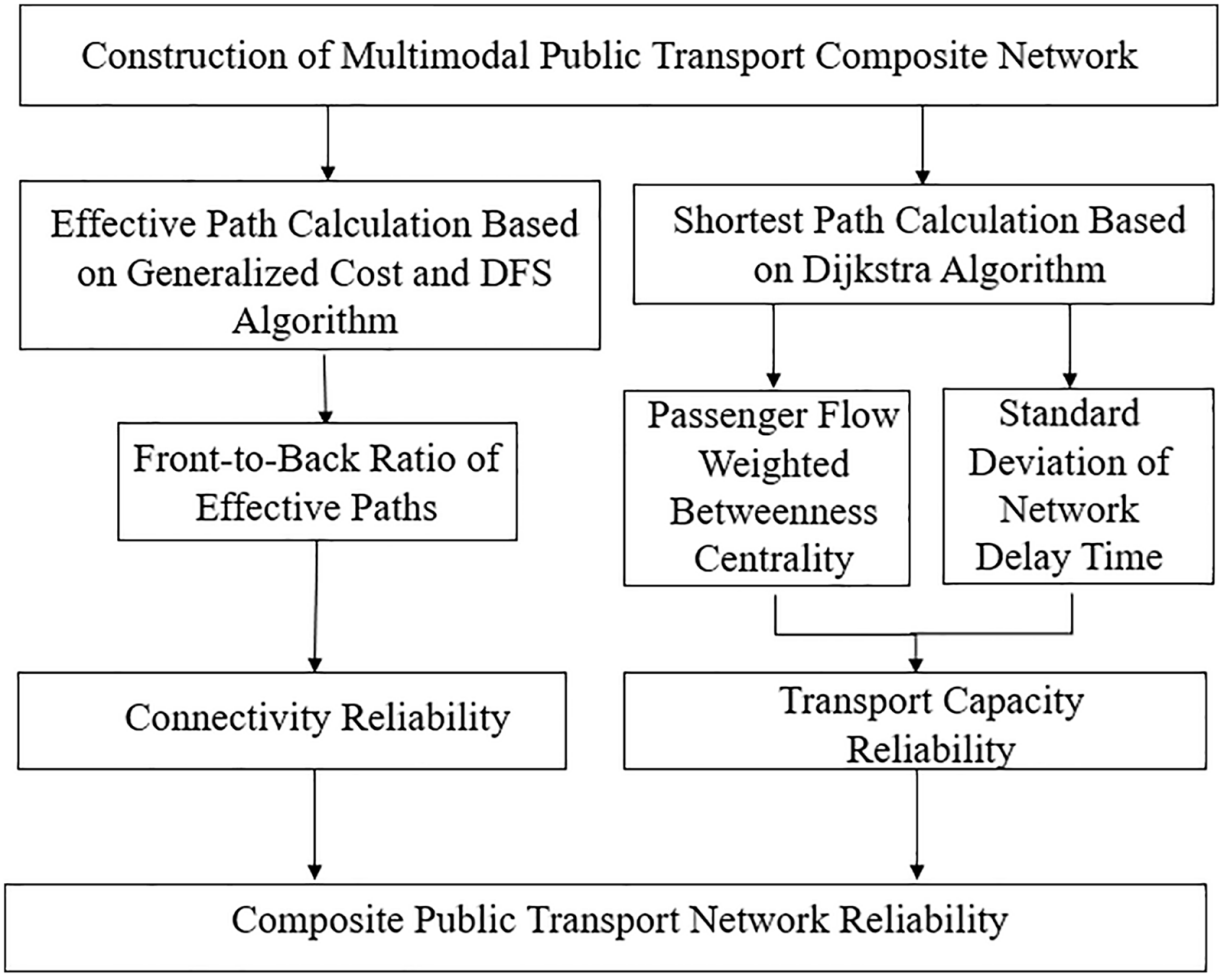
Flowchart of reliability measurement for urban public transport composite networks.

The detailed process for measuring the reliability of urban public transport composite network is illustrated in Fig 1.

Firstly, a multimodal composite network is constructed by integrating urban rail and bus systems into a unified transport network structure. Then, a depth-first search (DFS) algorithm based on generalized cost and relative threshold is used to traverse the network and compute the ratio of effective OD paths before and after disruptions, from which the connectivity reliability index is derived. In addition, the Dijkstra algorithm is employed to calculate the shortest paths between OD pairs, while estimating node-level passenger flow and delay times to assess the transport capacity reliability of the network.

- Generalized travel cost function for network paths

The generalized travel cost index is used not only for path optimality judgment but also for determining the number of effective OD paths, which further reflects the level of network connectivity reliability. The generalized travel cost for a path in the composite network consists of two components: segment travel cost and node transfer cost.

The segment travel cost incorporates congestion-adjusted travel time and dwell time, and is formulated as:


$$\begin{array}{c} \;\;T_{ij}^{k}=\left[\sum {\mathrm{\backslash nolimits}}_{e\epsilon E_{m}}t_{em}(\text{1}+Y(x_{em}))+\sum {\mathrm{\backslash nolimits}}_{i}P_{mi}\right]\delta_{e,k}^{i,j}+\left[\sum {\mathrm{\backslash nolimits}}_{e\in E_{b}}t_{eb}(\text{1}+Y(x_{eb}))+\sum {\mathrm{\backslash nolimits}}_{i}P_{bi}\right]\delta_{e,k}^{i,j} \end{array}$$ (9)

Where $Y(x_{em})$ and $Y(x_{eb})$ are the congestion coefficients for rail and bus, $t_{em}$ and $t_{eb}$ are the segment travel times, $P_{mi}$ and $P_{bi}$ are the dwell times at rail and bus stops, respectively.

Transfer walking time depends on corridor length L, pedestrian speed, and facility comfort; waiting time is typically modeled as half the headway H. To reflect passengers’ sensitivity to transfer time, transfer penalty coefficients $\varphi_{\text{1}}$, $\varphi_{\text{2}}$ are introduced to account for perceived costs. The formulas are:


$$E_{ij}^{k}=\varphi_{\text{1}}t_{w}+\varphi_{\text{2}}t_{d}$$ (10)

$${\;E}_{ij}^{k}={\left[\varphi_{\text{1}}\sigma \frac{L}{\overset{―}{v}}+\varphi_{\text{2}}\left[P_{d}\frac{\text{3}H}{\text{2}}+(\text{1}-P_{d})\frac{H}{\text{2}}\right]\mathrm{\backslash rightleft}(n_{e,k}^{i,j}\right)}^{\gamma_{\text{1}}}+\varphi_{\text{3}}t_{f}{\left(n_{e,k}^{i,j}\right)}^{\gamma_{\text{2}}}$$ (11)

$$n_{e,k}^{i,j}=\sum_{e\in E}\delta_{e,k}^{i,j}$$ (12)

Where $\overset{―}{v}$ is walking speed(km/h), σ is comfort coefficients, $\left(n_{e,k}^{i,j}\right)$ denotes the cumulative number of transfers experienced by passengers along path *k* between nodes *i* and *j*. It reflects the continuity of passenger travel within the composite network. The sharp increase in perceived transfer cost caused by the cumulative number of transfers can be represented by an amplification factor  γ.

The generalized travel cost for a passenger from node *i* to *j* along path *k* is:


$$c_{ij}^{k}=T_{ij}^{k}+E_{ij}^{k}$$ (13)

- Effective path determination rules

In practical operations, passengers tend to select the path(s) with the lowest generalized travel cost. If all available paths exceed their cost expectations, they may switch to alternative transport modes. Effective path screening is foundational for passenger flow assignment and has been widely studied and applied. Due to high accessibility, multimodal service levels, and complex transfer structures, the effective path constraints in a composite network are more intricate than in single-mode networks. Therefore, a relative threshold method is adopted, which selects a set of effective OD paths whose generalized cost does not exceed a multiple of the minimum cost path. The constraint condition is expressed as follows:


$$P_{ij}=\left\{\forall P_{ij}^{k}|(\forall s_{ij}^{m},s_{ij}^{n}\in P_{ij}^{k},s_{ij}^{m}\neq s_{ij}^{n})and(c_{ij}^{k}\leq c_{ij}^{min}(\text{1}+g))\right\}$$ (14)

Where $c_{ij}^{k}$ is the generalized travel cost of the *k*-th path from node *i* to node *j*, $c_{ij}^{min}$ is the minimum cost among all paths for OD pair (*i, j*), g is the relative threshold parameter. The default value of *g* is 1.5.

- Effective path search algorithm

Urban public transport composite network exhibit high complexity due to many nodes, edges, and transfer links. The depth-first search (DFS) algorithm is well-suited for path discovery in such networks. This study employs a relative-threshold-based DFS algorithm to identify all effective paths between OD pairs. The detailed steps are as follows:

Step 1: initialize variables in the composite network; set the relative threshold value g.

Step 2: import failed nodes and remove corresponding nodes and edges from the network to update its topology.

Step 3: use the shortest path algorithm to calculate the minimum cost path for each OD pair.

Step 4: set the current node r as the root and traverse neighboring nodes s. If the path cost from r to s satisfies the threshold criterion in Equations (1–14), set s as the new current node and go to Step 5; otherwise, proceed to Step 7.

Step 5: if node s is not the destination, return to Step 4; otherwise, proceed to Step 6.

Step 6: record the valid path.

Step 7: backtrack to the previous level. If the root node r has not yet been revisited, return to Step 4. Otherwise, discard the path and end the traversal.

### Analysis method for reliability evolution of urban public transport composite network

#### Coupled map lattice evolution model.

The cascading failure process of the composite network can be modeled using the Coupled Map Lattice (CML) [27], a dynamic system framework capable of capturing complex spatiotemporal behaviors in nonlinear systems. The CML model is traditionally utilized to capture the state evolution of nodes through local coupling rules. In this study, we extend the CML framework to model not only node states but also link states [28], which is justified by the tightly coupled nature of nodes and links in composite public transport networks. The state of a link (e.g., passenger flow, delay) is directly influenced by the functional state of its adjacent nodes, while link failures also propagate disruptions back to nodes—a feedback mechanism that aligns with the dynamic coupling principles of CML. This bidirectional state dependency makes it reasonable to incorporate both nodes and links within the same CML-based dynamical system, thereby enabling a more holistic simulation of cascading failures across the network. In this model, node state variables are continuous, while time and space are discrete. The state variable at time t is defined as:


$$\begin{array}{c} x_{i}(t+\text{1})=(\text{1}-\varepsilon )f\left(x_{i}(t)\right)+\varepsilon \sum {\mathrm{\backslash nolimits}}_{j=\text{1}}^{n}\frac{ϝx_{j}(t)}{k_{i}} \end{array}$$ (15)

Building upon the classical CML framework, the measurement index of transport capacity reliability is introduced and the scalar state variable is extended to a multi-dimensional vector. A dynamic coupling mechanism is proposed as:


$$\begin{array}{c} x_{i}(t+\text{1})=(\text{1}-\varepsilon )f\left(x_{i}(t)\right)+\varepsilon \sum {\mathrm{\backslash nolimits}}_{i=\text{1}}^{N}\frac{C_{B}^{W}\left(v_{i}\right)}{C_{B}^{max}}\cdot \frac{ϝx_{i}(t)}{k_{i}} \end{array}$$ (16)

$$\varepsilon =\sum_{i=\text{1}}^{n}\frac{k_{i}}{n}$$ (17)

Where $x_{i}(t)$ is the state variable of network node *i* at time *t*, $C_{B}^{max}$ is the maximum passenger flow–weighted betweenness centrality in the entire network, $k_{i}$ is the degree of node *i*, n is the total number of nodes in the network, ϝ is the function representing the coupling form between network nodes, which may be linear coupling  ϝ(x)=x or nonlinear coupling  ϝ(x)=g(x), where  g(x) is a nonlinear function.

Nodes with higher degree exert greater influence on their neighborhoods, reflecting the dominant role of hub nodes during cascading failures. By incorporating passenger flow–weighted betweenness, the functional significance of nodes is accurately represented, enhancing the model’s ability to capture key nodes in failure propagation.

#### Identification of node and edge states.

1. Node state

Attack strategies are used to simulate station-level failures. General attacks lead nodes to transition from normal to saturated states, while severe attacks result in complete failure. According to the passenger flow redistribution assumption, flow from saturated or failed nodes is rerouted to nearby normal nodes or links. In practice, travelers prefer proximate alternatives to minimize travel cost.

Let an external disruption be applied to node v at time t, then its state variable is expressed as:


$$\begin{array}{c} S_{i}(t+\text{1})=\left|\left(\text{1}-\varepsilon_{\text{1}}\right)f\left(S_{i}(t)\right)+\varepsilon \sum {\mathrm{\backslash nolimits}}_{j=\text{1},j\neq i}^{N_{G}}\frac{F_{ij}f\left(S_{i}(t)\right)}{I_{i}}\right| \end{array}$$ (18)

$$S_{i}(t)=[S_{i}^{struct}(t),S_{i}^{cap}(t)]$$ (19)

$$S_{i}^{cap}(t)=\frac{C_{B}^{W}\left(v_{i}\right)}{C_{B}^{max}}\cdot \frac{M^{threshold}}{M_{i}^{t}}$$ (20)

Here, $S_{i}(t+\text{1})$ and $S_{i}(t)$ denote the state variables of node  i  at time t+1 and t, respectively. Assume that:


$$\left\{ \begin{array}{c} When\;\text{0}<S_{i}(t)<\text{1},t\text{he}\;\text{node}\;\text{is}\;\text{in}\;\text{a}\;\text{normal}\;\text{state}\;\text{and}\;\text{not}\;\text{under}\;\text{attack};\\ When\;S_{i}(t)=\text{1},the\;node\;is\;in\;a\;saturated\;state\;due\;to\;a\;general\;attack;\\ \text{When}\;S_{i}(t)>\text{1},\text{the}\;\text{node}\;\text{is}\;\text{in}\;\text{a}\;\text{failed}\;\text{state}\;\text{due}\;\text{to}\;\text{a}\;\text{severe}\;\text{attack}. \end{array}\right.\;$$ (21)

Where $S_{i}(t)$ *is the* state variable of node  i at time t, $S_{i}^{struct}(t)\in [\text{0},\text{1}]$ *denotes* structural state of node *i,* $S_{i}^{cap}(t)\in [\text{0},\text{1}]$ *denotes* transport capacity state of node *i,* $M^{threshold}$ *is the* threshold of historical delay standard deviation, ε *is the* coupling strength between network nodes, $N_{G}$ is the number of nodes in the largest connected component of the network, $F_{ij}$ is the passenger flow on edge $e_{ij}$ (persons/hour), $I_{i}$ is the passenger flow at node  i (persons/hour).

Based on Equations (17–20), the state of a node within the composite network at a given time instant can be characterized. The absolute value operator in the equation ensures the non-negativity of the node state variable. Due to the inherent chaotic nature of traffic flow, it can be effectively modeled using the Chaotic Logistic Map, defined as  f(s)=4s(1−s),0≤s≤1. This mapping function  f  enables the representation of each node in the composite network as a chaotic dynamical system, thereby facilitating the simulation of localized dynamic behaviors observed in practical bus composite networks.

2. Edge state

In urban public transport composite networks, the states of nodes and edges are mutually dependent, yet most existing studies focus primarily on node failures while overlooking edge conditions. Research indicates that under general attacks, saturated nodes may retain edge connectivity but are unable to transport passengers; under severe attacks, failed nodes cause their associated edges to fail, and the failure of any connected node will likewise result in edge failure. In practical operations, unexpected incidents leading to service interruptions often trigger metro or bus suspensions, shortened routes, or skip-stop operations, thereby inducing partial network failures. Consequently, the state of edges in urban public transport composite networks is jointly affected by both their own conditions and the states of the connected nodes, and can thus be expressed as:


$$\begin{array}{c} y_{ij}(t+\text{1})=\left\{\left|(\text{1}-\varepsilon )f\left(y_{ij}(t)\right)+\frac{\varepsilon }{\text{2}}\left[\sum {\mathrm{\backslash nolimits}}_{p=\text{1},p\neq i,j}^{N}\frac{F_{ip}f\left(y_{ip}(t)\right)}{I_{i}}+\sum {\mathrm{\backslash nolimits}}_{q=\text{1},q\neq i,j}^{N}\frac{F_{jq}f\left(y_{jq}(t)\right)}{I_{j}}\right],S_{i}(t),S_{j}(t)\right|\right\} \end{array}$$ (22)

$$y_{ij}(t)=[y_{ij}^{struct}(t),y_{ij}^{cap}(t)]$$ (23)

$$\left\{ \begin{array}{c} \text{0}\leq y_{ij}(t)<\text{1},the\;edge\;e_{ij}\;\text{is}\;\text{in}\;\text{a}\;\text{normal}\;\text{state},\text{unaffected}\;\text{or}\;\text{under}\;\text{mild}\;\text{attack}\\ y_{ij}(t)\geq \text{1},\;the\;edge\;e_{ij}\;\text{is}\;\text{in}\;\text{a}\;\text{failed}\;\;\text{state}\;\text{due}\;\text{to}\;\text{a}\;\text{severe}\;\text{attack} \end{array}\right.\;$$ (24)

Where $y_{ij}(t)$ denotes the state variable of edge $\;e_{ij}$ at time *t*, $y_{ij}^{struct}(t)\in [\text{0},\text{1}]$ denotes the structural state (normal/failure), $y_{ij}^{cap}(t)\in [\text{0},\text{1}]$ denotes the dynamic weight based on passenger flow $F_{ij}\;$ and delay time, $F_{ip\;}and\;F_{jq}$ denote the passenger flows on edges ${\text{e}}_{\text{ip}}\;\text{and}\;{\text{e}}_{\text{jq}}$ respectively (persons/hour), $I_{i}\;and\;I_{j}\;$ denote the passenger flows at nodes i  and j respectively (persons/hour).

The state of edge $e_{ij}$ is influenced not only by its own condition but also by the states of nodes i  and  j. Let $S_{i}(t)$ and $S_{j}(t$ denote the states of nodes  i  and  j at time t, respectively. If $\;S_{i}(t)>\text{1}\;$ or $S_{j}(t)>\text{1}$, then $y_{ij}(t+\text{1})>\text{1}$, indicating that if either of the connected nodes is in a failed state, the corresponding edge also enters a failed state. Furthermore, if $\text{0}<S_{i}(t)<\text{1}\;$ and $\text{0}<S_{j}(t)<\text{1}$, but $y_{ij}(t)>\text{1}$, then $y_{ij}(t+\text{1})>\text{1}$ still holds, implying the persistence of edge failure under the influence of prior edge failure and non-failed node states. Based on these relationships, the state of an edge in the composite network at a given time can be determined.

#### Passenger flow redistribution rules.

Following an attack, the states of nodes and edges within the composite network may undergo significant changes. The resulting passenger flow redistribution can be categorized into three scenarios: node oversaturation, node failure, and edge failure. Set isolated nodes and edges resulting from node or edge failures to a failed state, and remove the transferred passenger flow. The three types of passenger flow redistribution rules are as follows.

- Redistribution rule for node failure

Step 1: identify node *i* in a failed state, indicating that it is no longer operational. Passengers are unable to travel to, from, or through this node via any mode within the composite transit system.

Step 2: remove node  i and all edges $e_{ik}$ connected to it from the network.

Step 3: redistribute the passenger flow $I_{i}$ of node i  (defined as the sum of passenger flows on all edges connected to node *i*) to all neighboring nodes *j* that remain in a normal operating state.

Step 4: update the passenger flows on edges $e_{jl}$ connected to node *j* as follows:


$$F_{jl}^{\prime }=F_{jl}+\Delta F_{jl}$$ (25)

$$\Delta F_{jl}=\frac{F_{ij}\cdot F_{jl}}{I_{i}-F_{ij}}$$ (26)

Step 5: determine whether all nodes have been traversed. If so, terminate the process; otherwise, repeat Steps 1–4.

Lows:

- Redistribution rule for node oversaturation

Step 1: identify node *i* that is in a saturated state, meaning that although the node remains operational, no additional passengers can enter it.

Step 2: remove node *i* from the node set, but retain all adjacent edges to preserve the structural integrity of the network routes.

Step 3: redistribute the passenger flow of the saturated node *i* to its adjacent normal edges $e_{ik}$ and $e_{jl}$, where the flow on edge $e_{ik}\;$ is updated as follows:


$$F_{ik}^{\prime }=F_{ik}+\Delta F_{ik}$$ (27)

$$\Delta F_{ik}=\frac{F_{ij}\cdot F_{ik}}{I_{i}-F_{ij}}$$ (28)

Step 4: change the passenger flow on edge $\;e_{jl}$ as same as that on edge $\;e_{ik}$.

Step 5: Determine whether the traversal is complete. If so, terminate the process; otherwise, repeat Steps 1–3.

- Redistribution rule for edge failure

Step 1: identify failed edge $e_{ij}$ in the network, which is no longer operational. This results in route disruption within the composite network and may cause service interruption for both transit modes.

Step 2: remove the failed edge ${\;e}_{ij}$ from the network.

Step 3: redistribute the passenger flow previously carried by edge $\;e_{ij}\;$ to adjacent normal edges $e_{ik}\;and$ $e_{jl},\;$ with the flow on edge $e_{ik}\;$ updated as follows:


$$F_{ik}^{\prime }=F_{ik}+\Delta F_{ik}$$ (29)

$$\Delta F_{ik}=\frac{F_{ij}\cdot F_{ik}}{I_{i}-F_{ij}}$$ (30)

Step 4: change the passenger flow on edge ${\;e}_{jl}$ as that on edge $e_{ik}$.

Step 5: determine whether the traversal is complete. If so, terminate the process; otherwise, repeat Steps 1–4.

The passenger flow redistribution mechanism ensures that total network flow remains conserved during each iteration. Mathematically, the inflow and outflow of each node satisfy,thereby preventing artificial gain or loss of passengers in the simulation.

## Case study

1. Construction of urban public transport composite network

**Fig 2:**
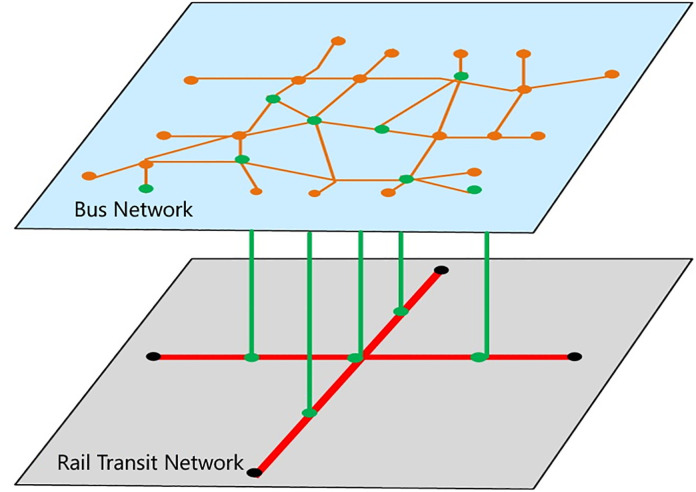
Schematic diagram of the urban composite bus network in the core area of Nanguan District.

This study selects the core area of Nanguan District in Changchun City as the case study area. There are three urban rail transit lines within this area: Metro Line 1, Metro Line 2, and Light Rail Line 3. 16 rail transit stations are in the study area, with 8 stations on Line 1, 4 on Line 2, and 5 on Line 3. There are two transfer stations: Jiefang Avenue Station, which serves as an interchange between Lines 1 and 2, and Weixing Square Station, which connects Lines 1 and 3, as illustrated in Fig 2. In addition, there are 105 bus routes, covering 77 bus stops within the composite network. Although this area features highly integrated rail transit and conventional bus services, a complete network struc-ture, and dense passenger flows—effectively representing the typical operational characteristics and failure modes of a composite public transport network, it does not cover the entire city network. This limitation may somewhat affect the generalizability of the research conclusions. Future work will expand to a more comprehensive urban road network to further enhance the representativeness and applicability of the study.

Based on rail transit and conventional bus route information obtained via the Gaode Map API, Python web crawling techniques were employed to extract inter-station connectivity data. The information was then georeferenced and annotated on a panoramic map to construct the base topology of the urban transport network, as illustrated in Fig 2.

Composite node identification rules were set as follows: if the average daily transfer passenger flow between a rail transit station and a bus stop exceeds 1000, or if they share three or more bus routes, the rail transit station and bus stop are considered as a composite node. Using this rule, 12 composite nodes, such as Weixing Square and Jiefang Avenue, were identified, as listed in Table 1. This function-oriented node aggregation approach can accurately capture the intermodal coordination relationships within the multimodal transit system, providing a realistic network foundation for cascading failure analysis.

**Table 1:** Example of composite nodes (Metro Line 1).

Metro Line 1 – Subway Stations								
Subway Station	Victory Park	People’s Square	Liberation Avenue	Northeast Normal University	Workers’ Square	Prosperity Road	Satellite Square	(Municipal Government
Bus Station	Beijing Avenue Victory Park	Chongzhi Mall Banruo Temple Min’an Road People’s Square	Liberation Avenue South Liberation Avenue North Liberation Avenue	Northeast Normal University Scholar’s Bookstore	South Lake Avenue Workers’ Square Nanhu Swimming Area	Huning Road Prosperity Road	Weixing Guangchang South Entrance Weixing Guangchang North Entrance Expressway Coach Terminal	Provincial Library Municipal Party Committee Municipal Government

2. Calculation of connectivity reliability

Based on the generalized travel cost function, effective path determination rules, and path search algorithms, the connectivity reliability of the composite network can be evaluated.

In the composite network, the generalized travel cost of a path consists of two components: segmental travel cost and node transfer cost. Firstly, using data from the Gaode Map API, the travel time and distance between stations are obtained by analyzing inter-station distances and durations. These values are then used to construct a weighted adjacency matrix that reflects the connectivity of the bus network. This adjacency matrix captures the link relationships among nodes and serves as the input for subsequent calculations. Using Equations (19) through (1–13), the generalized travel costs for all feasible paths are computed in MATLAB. A subset of the path travel costs is presented in Table 2. By analyzing these path costs, the connectivity reliability of the entire network can be further assessed.

**Table 2:** Generalized travel cost of paths in the composite network.

Path ID	Origin	Destination	Segment cost (min)	Transfer cost (min)	Total cost (min)
1	Victory Park	Yitong River	18	4	22
2	Jiefang Avenue	Northeast Normal University	8	0	8
3	Jilin Univ. (Nanling)	Yatai Overpass	16	2	18
4	Linhe Street	Dongwan Peninsula A Zone	16	0	16

Effective path filtering in the composite network refers to identifying travel paths within the network topology that satisfy the constraints of the generalized travel cost function. Based on the effective path determination rules and the applied search algorithm, the relative threshold method is used to extract valid paths, as summarized in Table 3.

**Table 3:** Set of effective routes.

Path ID	Node Sequence (Origin → … → Destination)
1	GONGNONG Square→NANHU Avenue→XINWENHUA Newspaper Office→HUNING Road→→Prosperity Road→North Entrance of Satellite Square→Changchun University)→University of Science and Technology (Bus-to-Metro Transfer)→WEIGUANG Street→Convention and Exhibition Center→WEIXING Road

3. Calculation of connectivity reliability

By assigning passenger flows to the study composite network, the dynamic interaction between network structure and passenger demand can be simulated. According to statistical data released by the Changchun Municipal Transportation Bureau, in 2023, the average daily passenger volume of Changchun’s conventional bus system was 2 million trips and the average daily passenger flow of rail transit is 500,000 trips. The allocation of average daily passenger flows for both transit modes to individual nodes and links follows a defined rule: firstly, the number of public transit routes passing through each network edge (i.e., the multiplex coefficient of the edge) is counted; then, the proportion of each edge’s multiplex coefficient relative to the total is calculated; by multiplying this proportion with the total daily ridership of each transit mode, the passenger flow on each edge is obtained; and finally, the passenger flow of a node is determined by summing the flows of all edges connected to it.

The passenger flow at each node is calculated accordingly, and the proportion of the top ten nodes ranked by passenger flow is shown in Table 4.

**Table 4:** Passenger flow share ranking in the composite network.

Node Name	Flow Share	Rank
Satellite Square	0.25	1
Jiefang Avenue	0.22	2
Jiefang Avenue	0.14	3
Prosperity Road	0.10	4
Yatai Avenue	0.05	5
Victory Park	0.05	6
……	……	……

The actual passenger flow at a given station *v* is statistically obtained and used as the weighting factor in the calculation of flow-weighted indicators. The passenger flow at each network node reflects the relative importance of that node in terms of traffic demand. Based on this, node betweenness is calculated using Dijkstra’s shortest path algorithm. The results of the transport capacity reliability analysis for the composite network are presented in Table 5.

**Table 5:** Passenger flow-weighted betweenness centrality in the composite network.

Node Name	Flow-Weighted Betweenness
Satellite Square	0.85
Jiefang Avenue	0.82
People’s Square	0.72
Yatai Avenue	0.70
Fanrong Road	0.55
……	……

Table 5 presents the transport capacity reliability indicators for individual stations within the network. High-reliability stations such as Weixing Square (0.80), Jiefang Avenue (0.78), and Renmin Square (0.72) demonstrate strong resilience in maintaining transport operations even under network disruptions. These stations are located at critical positions within the network, exhibiting strong connectivity and redundancy. Due to their higher reliability, these nodes are more capable of sustaining stable passenger service during emergencies or service failures. In contrast, low-reliability stations such as Fanrong Road (0.55) show relatively weak transport capacity reliability, indicating greater susceptibility to disruptions or failures. Such stations are located at the periphery of the network or have fewer direct connections to other nodes, resulting in a higher likelihood of service interruption or delay during network failures.

The average delay time at each station *v* is extracted by crawling real-time data from Gaode Map for the composite network. This delay time is used as an input for computing the Mahalanobis distance of each node. According to the transport capacity reliability evaluation model, a MATLAB program is developed to perform the reliability calculation. The results show that the standard deviation of network delay time is 0.1%, indicating a high level of temporal reliability and good operational stability within the network.

Overall, there are certain disparities in the transportation capacity reliability across different sites, indicating an uneven level of stability among them when facing network failures. High-reliability stations typically serve as major transport hubs or critical nodes, whereas low-reliability stations tend to be peripheral or less connected within the network.

4. Reliability evolution simulation
  - Simulation settings

Reliability evolution simulations are conducted with the following goals:

1) to investigate the impact of different attack strategies on the reliability of urban public transport composite network;
2) to examine the influence of network coupling strength on the network’s reliability.

The simulation scenarios are designed as shown in Table 6.

**Table 6:** Simulation scenario settings.

Scenario	Analytical objective	Attack strategy	Attack intensity
Ⅰ	Analyze the impact of different attack strategies on composite network	Random attack	Severe attack Mild attack
		Targeted attack	Severe attack Mild attack
		Traffic congestion	Severe attack Mild attack
		Sudden surge in passenger flow	Severe attack Mild attack
ⅠⅠ	Investigate the influence of network coupling strength on network reliability	Random attack	Mild attack
		Targeted attack	Mild attack

In this study, the disturbance intensity is quantitatively classified following the conventions of complex network robustness analysis (Albert et al., 2000; Berche et al., 2009; Zhang et al., 2020). Specifically, light attacks correspond to 10–20% node or link removal (or ≤20% capacity reduction), moderate attacks to 20–40%, and severe attacks to 40–50% removal or ≥50% capacity degradation at key nodes or links.This classification provides a consistent standard for comparing the effects of different attack strategies and facilitates the interpretation of cascading behavior under varying disruption levels.

**Fig 3:**
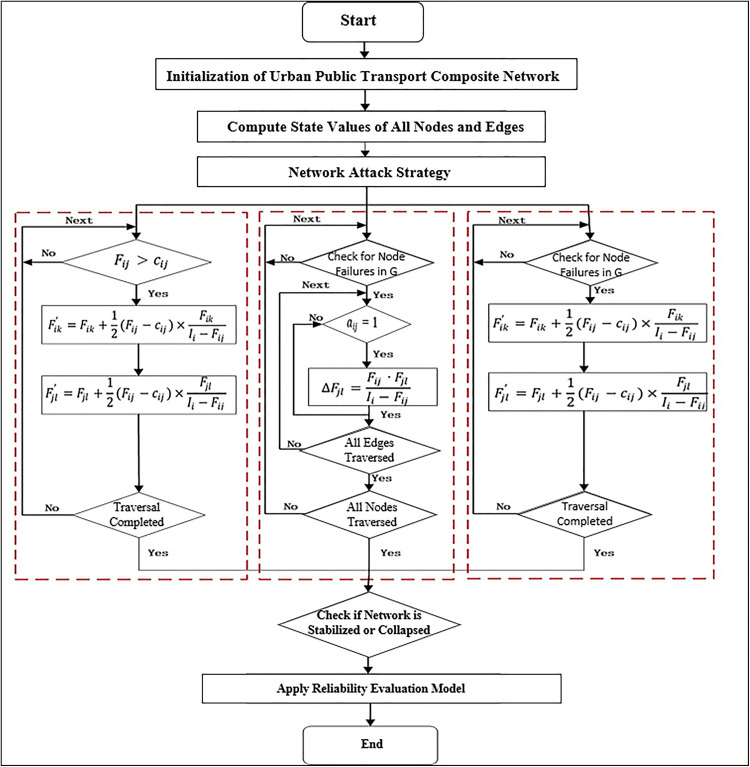
Simulation flowchart for reliability analysis of composite network.

The simulation process is as follows. Firstly, the initial state values of all nodes and edges in the composite network are set within the range [0,1]. Secondly, the failure processes of network nodes or links are simulated under various attack strategies. Finally, the reliability measurement model of the composite network is applied to calculate the reliability outcomes, followed by visualization of the results. The overall simulation process for evaluating the reliability of composite network is illustrated in Fig 3.

- Analysis of reliability evolution results
  Impact of different attack strategies on network reliability

Random attack strategies with varying intensities—severe attacks and mild attacks—are applied to the study composite network. The objective is to analyze how the network’s reliability indicators change as different numbers of nodes are affected by the attacks. In addition, targeted attack strategies are implemented with both severe and mild levels of disruption. Nodes are ranked in order of importance based on their degree centrality, as shown in Table 7. During targeted attacks, nodes are targeted according to their degree ranking. The impact on network reliability is then analyzed under varying degrees and quantities of node failures.

**Table 7:** Degree ranking in the composite network.

Node Name	Degree
Satellite Square (composite node)	14
People’s Square (composite node)	12
Culture Square (composite node	10
Jiefang Avenue (composite node)	9
……	……
Guangming Road (bus)	2
Nanhuancheng (bus)	2

**Fig 4:**
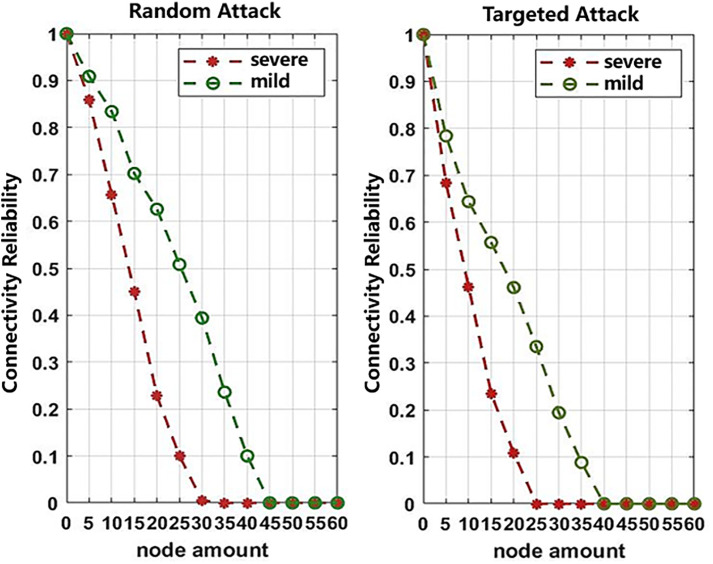
Connectivity reliability of the composite network under different attack strategies.

When the composite network is not under attack, all stations remain connected, and the network reliability is 100%. As shown in Fig 4, the effects of random attacks and targeted attacks on the composite network differ significantly. Under random attacks, the decline in network reliability is relatively gradual. Under mild attacks, network reliability drops to zero only after 70% of nodes fail, while under severe attacks, reliability reaches zero after 50% of nodes fail. This is because most randomly targeted nodes tend to be peripheral or isolated, thus exerting limited impact on overall network performance. In contrast, under targeted attacks, network reliability decreases sharply. Under severe attacks, reliability falls to zero after just 40% of nodes fail, while under mild attacks, it drops to zero after 60% node failures. This is due to the targeting of highly connected nodes, which substantially reduces the number of effective paths following passenger flow redistribution, resulting in near-total network paralysis. Moreover, severe attacks have a greater impact on network reliability, as they directly cause station failures and route disruptions. Mild attacks, on the other hand, have a lesser impact since the affected stations merely enter a saturated state and the network remains structurally connected.

**Fig 5:**
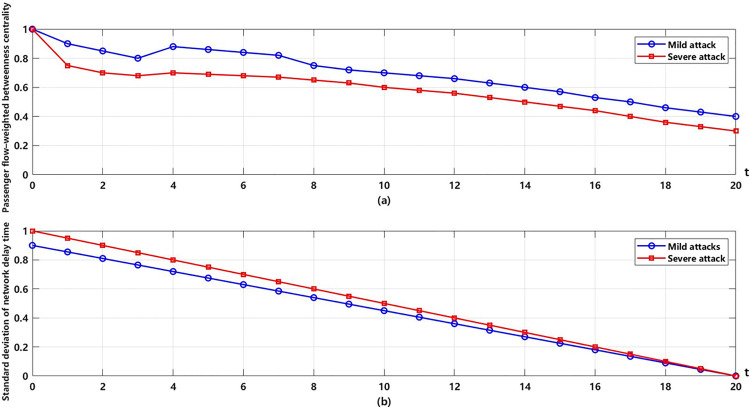
Transport capacity reliability of the composite network under different attack strategie.

Random attack strategies of both severe and mild intensity are applied to the composite network, to analyze how the transport capacity reliability indicators evolve as nodes are progressively compromised, as shown in Fig 5.

In Fig 5, the two charts reveal that under mild and severe attack scenarios, the standard deviation of network delay time exhibits a gradual downward trend over time. In both cases, the network’s flow-weighted betweenness centrality decreases during the initial iterations (from 0 to 4), with a steeper decline observed under severe attacks. Between iterations 4 and 6, it gradually recovers to around 0.8, indicating that the network’s capacity for passenger flow redistribution suffers significant disruption but also possesses a certain degree of resilience. These indicators reflect the network’s self-recovery capability facing disruptions. However, the final reliability level in both scenarios does not return to the original baseline, suggesting that the network incurs a degree of permanent damage.

Interestingly, the simulation results show a rebound in transport capacity reliability when the network damage exceeds approximately 30%. This phenomenon can be attributed to two main factors. First, the removal of certain redundant or congested nodes reduces flow concentration at bottlenecks, leading to a temporary improvement in the efficiency of flow redistribution. Second, after the elimination of these low-efficiency or structurally fragile components, the remaining network exhibits a more balanced load distribution, which mitigates overload pressure on critical nodes. This nonlinear effect is consistent with the “adaptive reconfiguration” mechanism observed in self-organized criticality of transport systems, where partial disruptions may lead to short-term performance gains before overall degradation dominates.

In summary, the results demonstrate the evolution of transport capacity reliability and connectivity reliability during the cascading failure process. Throughout the simulation, transport capacity reliability generally declines, though a slight increase in flow-weighted betweenness centrality is observed during iterations 2–6. This implies that the failure of certain redundant nodes may enhance overall network connectivity. The overall decline in connectivity reliability aligns with the cascading failure dynamics, indicating that the uniformity of passenger service across network nodes is relatively less affected by the cascading process. Transport capacity reliability proves to be more sensitive to disruptions than connectivity reliability. Under severe targeted attacks, the failure of just 20% of key nodes results in a 60% drop in transport capacity. Under the same level of attack intensity, the degradation rate of transport capacity is 1.8 times that of connectivity reliability. It highlights that the traditional evaluation systems focusing solely on connectivity significantly overestimate the actual service capacity of the network.

2) Impact of different coupling coefficients on the network reliability

Coupling coefficient *ε* represents the average number of coupling edges per node in the composite network. It characterizes the degree of interconnection between the different layers of the network and quantifies the strength of interactions between nodes in the network. A higher coupling coefficient indicates stronger interdependencies among nodes, resulting in greater susceptibility to disturbance propagation. Conversely, a lower coupling coefficient implies weaker inter-node influence and reduced transmission of disruptions. The value of coupling coefficient also significantly affects the extent to which failures can propagate through the network [29].

Based on calculations, the coupling strength of the composite network can be determined as 0.63.

**Fig 6:**
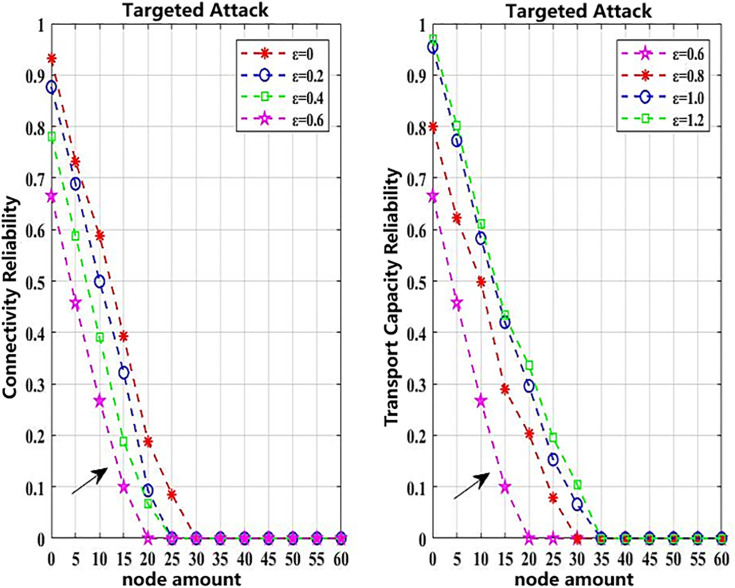
Impact of random attacks on network reliability under ε from 0 to 1.2.

**Fig 7:**
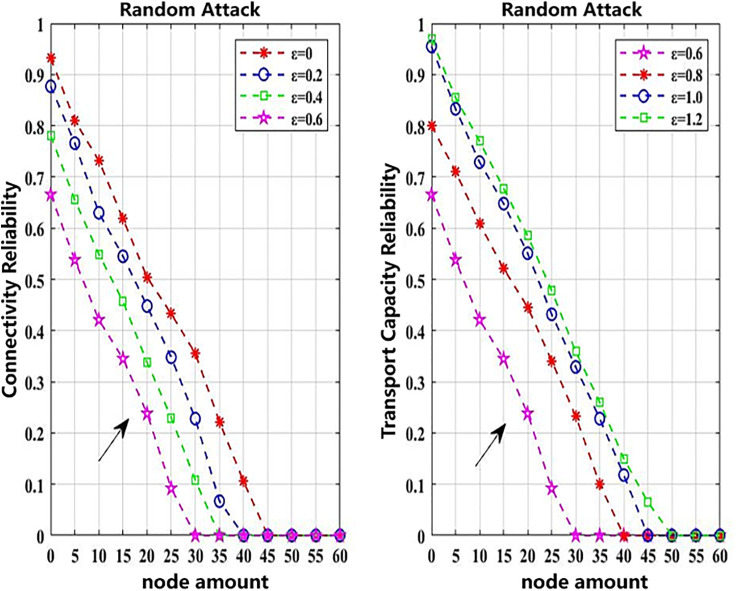
Impact of targeted attacks on network reliability under ε from 0 to 1.2.

The following simulation analyzes how variations in coupling strength affects network reliability. The simulation scenarios are set in Table 6, where random and targeted attacks of mild intensity are adopted as attack strategies. The objective is to observe the impact of different coupling strengths on the network reliability. When *ε* is set to 0, 0.2, 0.4, 0.6, 0.8, 1.0, and 1.2 respectively, the resulting effects on network reliability are shown in Figs 6 and 7. These results are derived from 1,000 simulation iterations, and the results are shown in Table 8.

**Table 8:** Statistical results of reliability indicators based on 1000 simulation runs.

Attack Scenario	Indicator	Standard Deviation (Std)	95% CI (Lower)	95% CI (Upper)
Random attack (mild)	Connectivity reliability	0.052	0.052	0.052
Random attack (severe)	Connectivity reliability	0.046	0.046	0.046
Intentional attack (mild)	Transport capacity reliability	0.061	0.061	0.061
Intentional attack (severe)	Transport capacity reliability	0.039	0.039	0.039

As shown in Figs 6 and 7, when *ε* ≤ 0.60, and the number of attacked nodes remains constant, the network reliability gradually decreases as the value of *ε* increases, regardless of the attack strategy. Since *ε* reflects the coupling strength between the urban rail transit and conventional bus networks within the composite system, a higher coupling strength (when *ε*  ≤ 0.60) leads to greater vulnerability and lower overall network reliability. However, when *ε* > 0.60, and the number of attacked nodes remains unchanged, further increases in *ε* lead to improved network reliability.

To mitigate the stochastic effects inherent in the CML model and the cascading failure process, 1000 independent simulation runs were conducted for each attack scenario. All simulations were performed under identical network topology and initial load conditions, while the selection of disturbed nodes and the corresponding load redistribution were allowed to vary randomly. As shown in Table 8, the final reliability results adopt the standard deviation (Std) and the 95% confidence interval (CI) as statistical indicators to ensure statistical significance and robustness.

## Conclusions

The study of reliability in urban public transport composite networks is critical to ensuring urban safety. The limitations of traditional study on public transport network reliability are the lack of dynamic transport capacity evaluation and the insufficient modeling of cascading failures. This article proposes a dual-layer measurement framework that integrates both connectivity reliability and transport capacity reliability. A novel dynamic evolution model based on the Coupled Map Lattice approach is constructed, which innovatively incorporates multidimensional parameters such as network topology, spatiotemporal passenger flow distribution, and node delay times. This model enables simulation-based exploration of the interaction mechanisms between node failures and passenger flow redistribution. By introducing transport capacity reliability, this study uncovers the dynamic phenomenon where localized node failures can temporarily enhance overall network efficiency through passenger flow redistribution. It’s an insight that static topological analysis cannot capture.

Taking the composite network of Nanguan District in Changchun City as a case study, the empirical results demonstrate that node passenger volume and delay time are key factors affecting network reliability. The findings reveal that passenger flow–weighted betweenness centrality significantly influences network performance, and that high coupling coefficients accelerate the cascading failure process. Compared to connectivity reliability, transport capacity reliability decays more rapidly. When the scale of network disruption exceeds 50%, connectivity reliability approaches zero, indicating a steep decline in the network’s structural integrity. Interestingly, within a 30% disruption threshold, transport capacity reliability exhibits a slight anomalous rebound, while beyond 50%, system reliability drops off sharply, confirming the critical importance of early-stage intervention to suppress fault propagation. Mid-stage optimization, such as rerouting or prioritizing repairs of critical nodes, may help sustain operational efficiency.

The proposed framework integrating timeliness and dynamic passenger flow reliability reveals the evolutionary patterns of urban transport systems under disturbances and provides a quantitative basis for route optimization, hub capacity planning, and timetable design. Future work can extend this approach to multimodal transportation systems to support integrated planning and emergency management. The research findings provide theoretical support for intelligent transportation systems. In the future, an expanded evaluation framework could incorporate multidimensional indicators such as passenger satisfaction and environmental carrying capacity. Furthermore, by integrating digital twin technology and reinforcement learning, real-time early warning systems can be developed. This would enable resilient simulation under complex scenarios, such as extreme weather events or large-scale public gatherings, ultimately facilitating a paradigm shift in public transport network reliability, from static assessment to dynamic monitoring and intelligent control.

## Supporting information

S1 Table Data support file. (XLSX)

S2 Code Simulation and analysis scripts for the reliability evolution model. (DOCX)
