# Supplementary material for: Measuring and evolution analyzing of the reliability in urban public transport composite network
Source: PLoS One. 2026 Jan 8;21(1):e0340590. doi: 10.1371/journal.pone.0340590 (PMC12782392; doi:10.1371/journal.pone.0340590)
Supplement: S2 Code — (DOCX) [file pone.0340590.s002.docx]

# Supplementary Code (S2): Reliability Modeling and Simulation

This supplementary document provides the complete and executable Python code used in this study. The code is organized into clearly defined sections corresponding to the methodological framework described in the manuscript.

## Section 1. Environment Setup and Data Loading

import pandas as pd

import numpy as np

import networkx as nx

import matplotlib.pyplot as plt

import random

df = pd.read_excel("S1_Data_FullyGenerated_Input.xlsx")

## Section 2. OD-based Composite Network Construction

nodes = sorted(set(df["Origin"]).union(set(df["Destination"])))

G = nx.Graph()

total_population = 2500000

avg_population = total_population / len(nodes)

for n in nodes:

G.add_node(n, population=avg_population)

for _, row in df.iterrows():

G.add_edge(

row["Origin"],

row["Destination"],

weight=row["Generalized Travel Cost (min)"],

time=row["In-Vehicle Travel Cost (min)"],

transfer=row["Transfer Cost at Nodes (min)"]

)

## Section 3. Connectivity Reliability Indicator

def connectivity_reliability(G):

if G.number_of_nodes() == 0:

return 0

largest_cc = max(nx.connected_components(G), key=len)

return len(largest_cc) / G.number_of_nodes()

## Section 4. Transport Capacity Reliability Indicator

def transport_capacity_reliability(G):

bc = nx.betweenness_centrality(G, weight="weight", normalized=True)

return sum(bc[n] * G.nodes[n]["population"] for n in G.nodes()) / len(G.nodes())

## Section 5. Delay Reliability Indicator

def delay_reliability(G):

X = np.array([[d["time"], d["transfer"]] for _,_,d in G.edges(data=True)])

if len(X) < 2:

return 0

mu = X.mean(axis=0)

cov = np.cov(X, rowvar=False)

inv_cov = np.linalg.pinv(cov)

dists = [(x-mu).T @ inv_cov @ (x-mu) for x in X]

return 1 / (1 + np.std(dists))

## Section 6. Cascading Failure Evolution Model (CML)

def CML_simulation(G, epsilon, attack_ratio, steps=30, targeted=False):

nodes = list(G.nodes())

state = {n: random.random() for n in nodes}

k = int(len(nodes) * attack_ratio)

if targeted:

targets = [n for n,_ in sorted(G.degree, key=lambda x: x[1], reverse=True)[:k]]

else:

targets = random.sample(nodes, k)

for n in targets:

state[n] += 1

reliability_curve = []

for _ in range(steps):

new_state = {}

for n in state:

f = lambda x: 4 * x * (1 - x)

neigh = list(G.neighbors(n))

coupling = np.mean([f(state[j]) for j in neigh]) if neigh else 0

new_state[n] = abs((1 - epsilon) * f(state[n]) + epsilon * coupling)

failed = [n for n,v in new_state.items() if v >= 1]

for n in failed:

G.remove_node(n)

new_state.pop(n)

state = new_state

reliability_curve.append(connectivity_reliability(G))

if G.number_of_nodes() == 0:

break

return reliability_curve

## Section 7. Simulation Experiments and Visualization

attack_ratios = np.linspace(0, 0.6, 8)

random_results = []

targeted_results = []

for a in attack_ratios:

random_results.append(np.mean(CML_simulation(G.copy(), 0.63, a, targeted=False)))

targeted_results.append(np.mean(CML_simulation(G.copy(), 0.63, a, targeted=True)))

plt.plot(attack_ratios, random_results, label="Random attack")

plt.plot(attack_ratios, targeted_results, label="Targeted attack")

plt.xlabel("Attack ratio")

plt.ylabel("Connectivity reliability")

plt.legend()

plt.tight_layout()

plt.show()

## Section 8. Output of Reliability Indicators

print("Connectivity reliability:", connectivity_reliability(G))

print("Transport capacity reliability:", transport_capacity_reliability(G))

print("Delay reliability:", delay_reliability(G))
